# Characterizing temporal genomic heterogeneity in pediatric low-grade gliomas

**DOI:** 10.1186/s40478-020-01054-w

**Published:** 2020-11-05

**Authors:** Margot A. Lazow, Lindsey Hoffman, Austin Schafer, Diana S. Osorio, Daniel R. Boué, Sarah Rush, Erin Wright, Adam Lane, Mariko D. DeWire-Schottmiller, Teresa Smolarek, Jared Sipple, Heather Taggert, Jaime Reuss, Ralph Salloum, Trent R. Hummel, Peter de Blank, Natasha Pillay-Smiley, Mary E. Sutton, Anthony Asher, Charles B. Stevenson, Rachid Drissi, Jonathan L. Finlay, Maryam Fouladi, Christine Fuller

**Affiliations:** 1 Brain Tumor Center, Cancer and Blood Diseases Institute, Cincinnati Children’s Hospital Medical Center, 3333 Burnet Ave, Cincinnati, OH 45229 USA; 2 Department of Oncology, Phoenix Children’s Hospital, Phoenix, AZ USA; 3 Department of Oncology, Nationwide Children’s Hospital, The Ohio State University, Columbus, OH USA; 4 Department of Pathology and Laboratory Medicine, Nationwide Children’s Hospital, The Ohio State University, Columbus, OH USA; 5 Department of Oncology, Akron Children’s Hospital, Akron, OH USA; 6 Department of Pediatrics, University of Cincinnati College of Medicine, Cincinnati, OH USA; 7 Department of Genetics, Cincinnati Children’s Hospital Medical Center, Cincinnati, OH USA; 8 Department of Pathology, Upstate Medical University, 750 E Adams St., Syracuse, NY 13210 USA; 9 Pediatric Neuro-Oncology, Nationwide Children’s Hospital, 700 Children’s Dr., Columbus, OH 43205 USA

**Keywords:** Pediatric low-grade gliomas, Genomics, Paired, Recurrence, Tumor evolution, *BRAF*, *CDKN2A*

## Abstract

Recent discoveries have provided valuable insight into the genomic landscape of pediatric low-grade gliomas (LGGs) at diagnosis, facilitating molecularly targeted treatment. However, little is known about their temporal and therapy-related genomic heterogeneity. An adequate understanding of the evolution of pediatric LGGs’ genomic profiles over time is critically important in guiding decisions about targeted therapeutics and diagnostic biopsy at recurrence. Fluorescence in situ hybridization, mutation-specific immunohistochemistry, and/or targeted sequencing were performed on paired tumor samples from primary diagnostic and subsequent surgeries. Ninety-four tumor samples from 45 patients (41 with two specimens, four with three specimens) from three institutions underwent testing. Conservation of *BRAF* fusion, *BRAF*^V600E^ mutation, and *FGFR1* rearrangement status was observed in 100%, 98%, and 96% of paired specimens, respectively. No loss or gain of *IDH1* mutations or *NTRK2*, *MYB*, or *MYBL1* rearrangements were detected over time. Histologic diagnosis remained the same in all tumors, with no acquired *H3K27M* mutations or malignant transformation. Changes in *CDKN2A* deletion status at recurrence occurred in 11 patients (42%), with acquisition of hemizygous *CDKN2A* deletion in seven and loss in four. Shorter time to progression and shorter time to subsequent surgery were observed among patients with acquired *CDKN2A* deletions compared to patients without acquisition of this alteration [median time to progression: 5.5 *versus* 16.0 months (*p* = 0.048); median time to next surgery: 17.0 months *versus* 29.0 months (*p* = 0.031)]. Most targetable genetic aberrations in pediatric LGGs, including *BRAF* alterations, are conserved at recurrence and following chemotherapy or irradiation. However, changes in *CDKN2A* deletion status over time were demonstrated. Acquisition of *CDKN2A* deletion may define a higher risk subgroup of pediatric LGGs with a poorer prognosis. Given the potential for targeted therapies for tumors harboring *CDKN2A* deletions, biopsy at recurrence may be indicated in certain patients, especially those with rapid progression.

## Introduction

Genomically-driven therapy is increasingly being incorporated into the treatment of pediatric low- grade gliomas (LGGs), the most common type of brain tumor in children [1, 31, 44]. While prognosis is excellent when a gross total resection can be achieved, young patients with incompletely resected and/or progressive disease pose therapeutic challenges and may experience a chronic, relapsing course, given relatively low durable response rates with standard chemotherapy and unacceptable long-term toxicity of irradiation [2, 14, 39]. Recent discoveries have provided valuable insight into the genomic landscape of pediatric LGGs at diagnosis, facilitating a shift in treatment strategy toward a molecularly targeted approach [31, 44]. An adequate understanding of the evolution of pediatric LGGs’ genomic profiles over time is critically important in guiding decisions about targeted therapeutics and diagnostic biopsy at recurrence.

Genetic aberrations within the mitogen-activated protein kinase (MAPK) pathway are prevalent in pediatric LGGs at diagnosis, resulting in activated downstream proliferation signaling and subsequent tumorigenesis [68]. Single driver genetic alterations have been consistently identified within specific histologic subtypes of pediatric LGGs, including *BRAF*-*KIAA1549* fusions in pilocytic astrocytomas [29, 54], *BRAF*^V600E^ point mutations in pleomorphic xanthoastrocytomas and gangliogliomas [17, 68], *FGFR1* duplications in diffuse astrocytomas [68], and *MYB* or *MYBL1* rearrangements in diffuse astrocytomas and angiocentric gliomas [4, 49, 58]. Expanded knowledge of the genetic landscape of LGGs has supported the growing investigation and utilization of molecularly targeted agents, such as MEK or BRAF inhibitors, for tumors with MAPK pathway alterations, especially at relapse or progression [5, 8, 23, 50].

Despite this reliable understanding of the biology underlying LGGs in children at diagnosis, little is known about their temporal genomic heterogeneity and whether they undergo genetic evolution following therapy and/or at recurrence. Genomic analyses of 10 paired adult LGGs revealed significant genetic variation between diagnosis and recurrence, including transformation to high-grade gliomas (HGGs) following chemotherapy in some patients [27]; however, molecular differences between adult and pediatric LGGs are well-recognized [28, 30, 51, 68], with relatively low risk of malignant transformation in children [9, 40], limiting generalizability of these findings to the pediatric patient population. A landmark report describing whole-genome sequencing of pediatric LGGs included two pairs of primary and recurrent tumors, which demonstrated identical genomic profiles [68], but, to our knowledge, no other studies have evaluated genetic changes of LGGs in children over time.

Emerging data suggest variable temporal genomic heterogeneity across other pediatric central nervous system (CNS) tumors. In medulloblastoma, molecular subgroup is conserved [48], but there is significant divergence in targetable mutations between diagnosis and recurrence [41]. Transcriptomic changes between matched primary and recurrent pediatric posterior fossa ependymomas have been reported, yet with relative preservation of copy number alterations [24]. A study of temporal genomic heterogeneity across 16 paired pediatric HGGs demonstrated conservation of certain key driver mutations at recurrence, but acquisition or loss of others [51].

Successful incorporation of molecularly targeted therapy and consideration of repeat biopsy at recurrence in pediatric LGGs demands an adequate understanding of how their genomic profiles evolve over time and following prior treatment. In this study, we characterize the temporal genomic heterogeneity of pediatric LGGs by comparing fluorescence in situ hybridization (FISH), mutation-specific immunohistochemistry (IHC), and/or targeted sequencing in paired tumor samples from primary diagnostic and subsequent surgeries.

## Materials and methods

### Clinical cohort

This retrospective study was performed at Cincinnati Children’s Hospital Medical Center (CCHMC), Nationwide Children’s Hospital, and Akron Children’s Hospital. The patient cohort was chosen based on the availability of adequate tumor specimens for testing from both primary diagnostic and subsequent surgeries (biopsy, resection, or autopsy), with a confirmed histologic diagnosis of LGG by neuropathology review (CF, DB). Samples from subsequent surgeries were only included for analysis if they occurred at least 1 month following the previous surgery. Patient tumor samples were preserved either as fresh-frozen or formalin fixed paraffin embedded (FFPE) tissue. To ensure adequate tumor content, hematoxylin and eosin (H&E) slides were reviewed from each frozen specimen, the initial cut of each FFPE block, and an additional cut of FFPE block after scrolls were obtained for DNA extraction. Clinical data, including age, sex, surgery details, and prior treatments, were abstracted from the patients’ electronic health records and subsequently de-identified. All patient tumor samples and clinical data were collected after informed consent was provided by patients or legal guardians through institutional review board approved protocols at the respective institutions.

### Fluorescent in situ hybridization (FISH)

FISH for the following relevant genetic alterations was performed on tumor specimens by the Department of Molecular Genetics at CCHMC: *BRAF* duplications or rearrangements, *FGFR1*, *MYB*, *MYBL1*, or *NTRK2* rearrangements, and *CDKN2A* deletions.

### Mutation-specific immunohistochemistry (IHC)

IHC staining for *H3K27M*, *BRAF*^V600E^, and *IDH1*-R132H mutations as well as *ATRX* loss was performed on slides cut from FFPE blocks of pediatric LGG samples using conventional methods [67].

### Targeted sequencing analysis

DNA extraction was carried out from frozen tissue using the Qiagen AllPrep DNA/RNA/miRNA Universal Kit following the manufacturer’s instructions. DNA from FFPE scrolls or core punches were isolated by suspending the paraffin scrolls in deparaffinization solution (Qiagen), followed by DNA extraction using the QIAamp DNA FFPE Tissue Kit. DNA quantification was conducted using the Quant-iT Picogreen or Qubit dsDNA assay (Thermo Fisher Scientific). Targeted DNA sequencing was performed on tumor specimens with adequate DNA for testing using the AmpliSeq 50 gene Focus Cancer Hotspot Panel V2 assay on the Illumina MiSeqDx instrument (Illumina, San Diego, CA). This panel, which requires 10–25 ng of genomic DNA and was validated on FFPE tissue, assessed mutations that include the following genes relevant to pediatric LGGs: *BRAF, FGFR1, FGFR2, FGFR3, HRAS, KRAS, NRAS, PTPN11, IDH1, IDH2,* and *TP53*, among others. Additionally, Foundation Medicine next generation sequencing (Foundation Medicine, Cambridge, MA) was performed on select tumor specimens and when available, relevant results from this testing were also included in the analysis.

### Statistical analysis

Continuous and categorical variables are described by median (range) and frequency (percent), respectively. The Wilcoxon Rank-Sum test and Fischer’s Exact test were used to assess for differences in age as well as interval systemic therapy and/or irradiation and World Health Organization (WHO) histologic grade, respectively, between patients whose tumors did or did not exhibit temporal genomic heterogeneity. Survival endpoints are described by the median time to event calculated using the Kaplan–Meier method. The Log-Rank test was used to evaluate potential associations between conservation or change in *CDKN2A* deletion status between diagnostic and recurrent tumor specimens with time to progression and time to subsequent surgery.

## Results

### Patient characteristics

A total of 94 primary diagnostic and subsequent surgical specimens from 45 pediatric patients with LGGs were included for analysis. Two tumor specimens were available for 41 patients, and three tumor specimens were available for four patients who underwent more than two surgeries (two of these four patients underwent four total surgeries, with identical histologic diagnoses confirmed on all four specimens for each patient, but only the latter three tumor specimens had adequate tissue for molecular testing). One other patient also underwent three total surgeries, but the time between his first two surgeries was less than 1 month (initial biopsy followed by gross total resection 1 week later), with identical histology and molecular testing on all specimens, so only specimens from the two surgeries more than 1 month apart were included for analysis. Two additional patients underwent a third neurosurgical resection due to radiographic or clinical concern for tumor progression, but pathology was not consistent with neoplasm (focal cortical dysplasia in one, reactive bone formation with dense fibrous tissue in the other), so these patients’ third specimens were not included in the analysis.

An overview of patient demographic and clinical characteristics as well as tumor histologic subtypes is shown in Table 1. Median time to second surgery was 19 months (range: 1.5–178 months), and median time from second to third surgery for those respective patients was 38.5 months (range: 1–118 months). Seventeen patients (38%) received systemic therapy; eight of these 17 patients received more than one successive systemic therapeutic regimen prior to second surgery due to recurrence/progression and/or treatment-related toxicity. Four patients (9%) received irradiation prior to their second surgery. Twenty-five patients (56%) did not undergo systemic therapy or irradiation prior to their second surgery; four of these patients did not have obvious clinical or radiographic concern for recurrence/progression, but a second surgery was undertaken to achieve maximal safe resection of residual tumor. Forty-two patients (93%) were alive at the time of last follow-up (median follow-up time of 87 months from diagnosis). Three patients passed away a median of 91 months (range: 71 to 128 months) from diagnosis [death was directly due to disease progression in one patient and due to unrelated causes in two patients (drug overdose, cardiogenic shock)].

**Table 1:** Overview of patient demographics and clinical characteristics

	Number of patients (%)
Gender	
Female	27 (60%)
Male	18 (40%)
Median age (years) at diagnosis (range)	5.8 (0.4–18.3)
Histologic diagnosis	
Pilocytic Astrocytoma	26(58%)
Pilomyxoid Astrocytoma	2 (4%)
Diffuse Astrocytoma	7 (16%)
Ganglioglioma	5 (11%)
Desmoplastic Infantile Ganglioglioma (DIG)	1 (2%)
Pleomorphic Xanthroastrocytoma (PXA)	1 (2%)
Angiocentric glioma	1 (2%)
Low grade glial or glioneuronal neoplasm, not otherwise specified (NOS)	2 (4%)
Extent of first surgical resection (at diagnosis)	
Biopsy	10 (23%)
Subtotal resection	24 (50%)
Gross total resection	11 (27%)
Median time (months) to first progression^a^ (Range)	13 (1.5–178)
Median time (months) to second surgery (Range)	19 (1.5–178)
Extent of second surgical resection	
Biopsy	2 (4%)
Subtotal resection	17 (38%)
Gross total resection	22 (49%)
Unknown	4 (9%)
Received systemic therapy prior to second surgery	17 (38%)
Received at least two systemic therapy regimens prior to second surgery	8 (17%)
Systemic therapy received (% of patients who received systemic therapy)	
Carboplatin with or without Vincristine	12 (71%)
Vinblastine	6 (35%)
Bevacizumab with or without Irinotecan	3 (18%)
Temozolomide	2 (12%)
Thioguanine, Procarbazine, Lomustine, and Vincristine	2 (12%)
Carboplatin or Cisplatin, Cyclophosphamide, and Etoposide	2 (12%)
Trametinib	1 (6%)
Everolimus	1 (6%)
Lenalinomide	1 (6%)
Vorinostat	1 (6%)
Rapamycin	1 (6%)
Received irradiation prior to second surgery	4 (9%)
Received no systemic therapy or irradiation prior to second surgery	25 (56%)
Underwent third surgery	4 (9%)
Median time (months) from second surgery to third surgery (range)	38.5 (1–118)
Extent of third surgical resection	
Biopsy	1 (25%)
Subtotal resection	3 (75%)
Received systemic therapy between second and third surgeries	2 (50%)
Systemic therapy received	
Temozolomide, then Vinblastine	1 (20%)
Avastin and Irinotecan	1 (20%)
Received irradiation between second and third surgery	0 (0%)
Received no systemic therapy or irradiation between second and third surgeries	2 (50%)
Underwent fourth surgical resection	2 (4%)
Median time (months) from third surgery to fourth surgery (Range)	45.5 (8–83)
Extent of fourth surgical resection	
Subtotal resection	1 (50%)
Autopsy	1 (50%)
Received systemic therapy between third and fourth surgeries	2 (100%)
Systemic therapy received	
Temozolomide, then Trametinib	1 (50%)
Lenalidomide, then Selumetinib, then Everolimus	1 (50%)
Received irradiation between third and fourth surgery	1 (50%)
Alive at time of last follow-up	42 (93%)
Median time (months) to last follow-up (Range)	87 (13–395)

^a^Time to first progression was defined as the time from diagnosis to a new medical or surgical intervention in response to clinical and/or radiographic concern for progression. This excludes four patients in the cohort who did not have obvious clinical or radiographic progression, but for whom a second surgery was undertaken to achieve maximal safe resection of residual tumor

### Histologic subtype and grade

Histologic subtype and grade were conserved in 100% (45 of 45) patients, including at third surgery, with no evidence of malignant transformation to HGG.

### Genomic findings

**Fig. 1:**
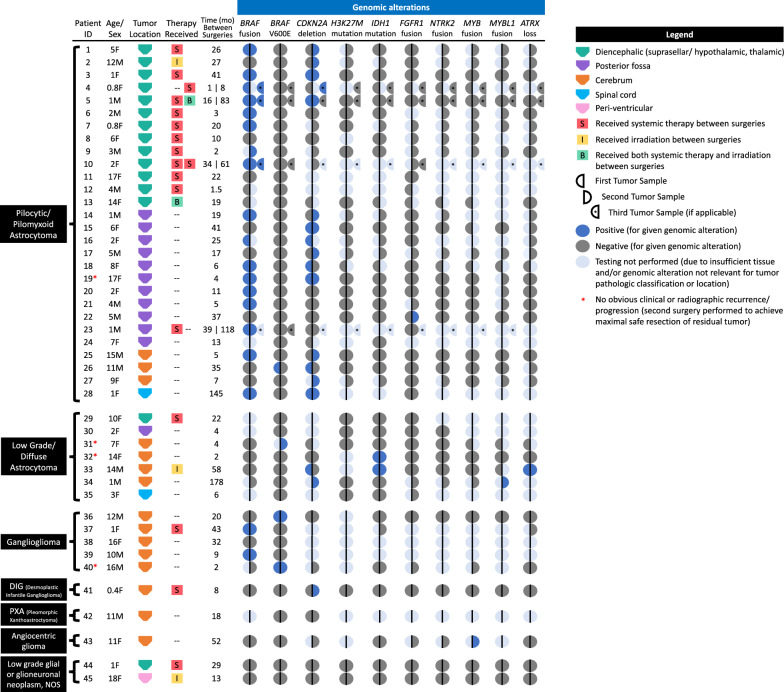
Genomic profiles of paired primary and recurrent and/or progressive tumor samples from 45 pediatric patients with LGGs analyzed in this study, grouped by histopathologic classification. Patients’ age (years), sex, tumor location, treatment received between primary and subsequent surgeries (systemic therapy [“S”], irradiation [“I”], or both [“B”]), and time interval (months) between respective surgeries are indicated. Each row of circles represents a tumor pair (or triplet) from an individual patient. The left half of a circle represents the primary diagnostic tumor sample, the right half represents the second subsequent surgical sample, and a third semicircle (with *) represents a third surgical sample if applicable. Dark blue, dark gray, and light blue semi-circles indicate positivity, negativity, or testing not able to be performed for the given genetic alteration, respectively

**Fig. 2:**
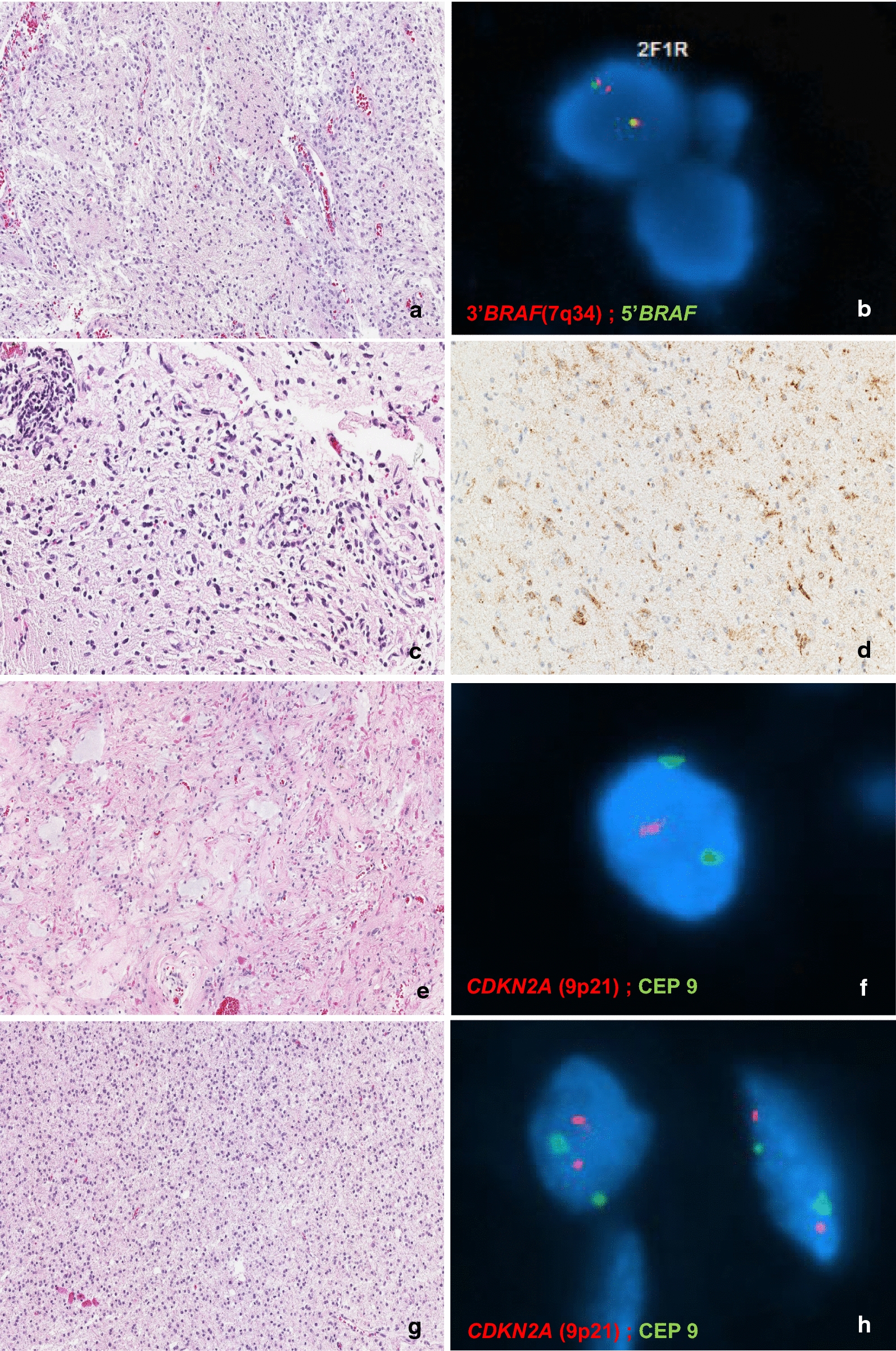
Histologic and molecular findings in paired samples from representative patients. **a** The pilocytic astrocytoma from Patient #18 retained a *BRAF* fusion. Note that the partial duplication of 3′BRAF (7q34) **b** is most commonly associated with the *BRAF*-*KIAA1549* fusion product. **c** The ganglioglioma from Patient #40 retained a *BRAF*^V600E^ mutation (**d** BRAF V600E IHC, 400x). **e** The pilocytic astrocytoma from Patient #2 acquired a hemizygous deletion (loss of one copy) of *CDKN2A* (**f**), while the diffuse astrocytoma (**g**) from Patient #33 lost this alteration (**h**). (2A, E, and G, H&E x100; 2C, x200)

A comparison of the genomic profiles of individual patients’ matched tumor specimens, grouped by histopathologic diagnosis, is illustrated in Fig. 1. Figure 2 provides images of the most commonly identified histologic and molecular (FISH and mutation-specific IHC) findings at diagnosis and second surgery from four representative patients. A summary of temporal genomic heterogeneity in the overall cohort and within specific histopathologic subgroups, as well as a breakdown of the number of patients who had specific genetic testing performed on paired specimens, is shown in Table 2 and detailed herein. Due to inconsistent tumor specimen availability and adequacy, it was not possible to perform all molecular tests on all paired tumor samples; therefore, in cases with sparse tissue, focused panels of genomic alterations were selected based on relevance to tumor histopathologic classification (e.g., prioritizing *IDH1* mutation testing for diffuse astrocytomas) and location (i.e., *H3K27M* IHC testing for tumors of midline location). Targeted sequencing was performed on paired tumor samples from 17 patients and relevant findings are incorporated in Fig. 1 and Table 2 and described below. There were no significant differences in age at diagnosis, previous systemic therapy and/or irradiation, or histologic WHO grade (I *versus* II) between patients whose tumors exhibited temporal genomic heterogeneity (n = 12) and patients whose tumors had completely conserved genomic profiles (n = 33; Table 3).

**Table 2:** Overview of temporal genomic heterogeneity

	*BRAF* fusion	*BRAF* V600E	*CDKN2A* deletion	*H3K27M* mut.	*IDH1* mut.	*FGFR1* fusion	*NTRK2* fusion	*MYB* fusion	*MYBL1* fusion	*ATRX* loss
Entire Cohort (all diagnoses, n = 45)										
Patients with paired tumor samples tested	34	44	26	21	28	28	17	16	12	18
Conserved										
Remained positive (S, I)	17 (S = 9, I = 1)	2 (S = 0, I = 0)	4 (S = 1, I = 0)	0	2* (S = 0, I = 1)	0	0	0	0	1^#^ (S = 0, I = 1)
Remained negative (S, I)	17 (S = 4, I = 3)	41 (S = 17, I = 5)	11 (S = 4, I = 1)	21 (S = 9, I = 3)	26 (S = 10, I = 2)	27 (S = 10, I = 4)	17 (S = 5, I = 3)	16 (S = 5, I = 3)	12 (S = 4, I = 2)	17 (S = 7, I = 3)
Changed										
Acquired (S, I)	0	0	7 (S = 3, I = 1)	0	0	1 (S = 0, I = 0)	0	0	0	0
Lost (S, I)	0	1 (S = 0, I = 0)	4^a^ (S = 1, I = 2)	0	0	0	0	0	0	0
By Histolopathologic diagnosis:										
Pilocytic or Pilomyxoid Astroyctoma (n = 28)										
Patients with paired tumor samples tested	23	28	19	13	15	19	9	10	7	10
Conserved										
Remained positive (S, I)	15 (S = 9, I = 1)	0	4 (S = 2, I = 0)	0	0	0	0	0	0	0
Remained negative (S, I)	8 (S = 2, I = 1)	27 (S = 14, I = 2)	6 (S = 2, I = 0)	13 (S = 6, I = 1)	15 (S = 7, I = 1)	18 (S = 8, I = 2)	9 (S = 3, I = 1)	10 (S = 3, I = 1)	7 (S = 2, I = 1)	10 (S = 5, I = 1)
Changed										
Acquired (S, I)	0	0	6 (S = 2, I = 1)	0	0	1 (S = 0, I = 0)	0	0	0	0
Lost (S, I)	0	1 (S = 0, I = 0)	3^a^ (S = 1, I = 1)	0	0	0	0	0	0	0
Diffuse Astrocytoma (n = 7)										
Patients with paired tumor samples tested	3	6	2	5	7	5	4	2	1	2
Conserved										
Remained positive (S, I)	0	0	0	0	2* (S = 0, I = 1)	0	0	0	0	1^#^ (S = 0, I = 0
Remained negative (S, I)	3 (S = 0, I = 1)	6 (S = 1, I = 1)	0 (S = 0, I = 0)	5 (S = 1, I = 0)	5 (S = 1, I = 0)	5 (S = 1, I = 1)	4 (S = 0, I = 1)	2 (S = 0, I = 1)	1 (S = 0, I = 0)	1 (S = 0, I = 1)
Changed										
Acquired (S, I)	0	0	0	0	0	0	0	0	0	0
Lost (S, I)	0	0	1 (S = 0, I = 1)	0	0	0	0	0	0	0
Ganglioglioma (n = 5)										
Patients with paired tumor samples tested	4	5	1	0	2	1	1	1	1	2
Conserved										
Remained positive (S, I)	2 (S = 1, I = 0)	2 (S = 0, I = 0)	0	0	0	0	0	0	0	0
Remained negative (S, I)	2 (S = 0, I = 0)	3 (S = 1, I = 0)	1 (S = 0, I = 0)	0	2 (S = 0, I = 0)	1 (S = 0, I = 0)	1 (S = 0, I = 0)	1 (S = 0, I = 0)	1 (S = 0, I = 0)	2 (S = 0, I = 0)
Changed										
Acquired (S, I)	0	0	0	0	0	0	0	0	0	0
Lost (S, I)	0	0	0	0	0	0	0	0	0	0
Other LGGs (n = 5)										
Patients with paired tumor samples tested	4	5	4	3	4	3	3	3	3	4
Conserved										
Remained positive (S, I)	0	0	0	0	0	0	0	0	0	0
Remained negative (S, I)	4 (S = 2, I = 1)	5 (S = 2, I = 1)	3 (S = 2, I = 0)	3 (S = 2, I = 1)	4 (S = 2, I = 1)	3 (S = 2, I = 1)	3 (S = 2, I = 1)	3 (S = 2, I = 1)	3 (S = 2, I = 1)	4 (S = 2, I = 1)
Changed										
Acquired (S, I)	0	0	1 (S = 2, I = 1)	0	0	0	0	0	0	0
Lost (S, I)	0	0	0	0	0	0	0	0	0	0

Results for the entire cohort (all histologic diagnoses) are shown together at the top, followed by results for each histologic diagnosis subgroup individually. The number of patients who had testing for a given genetic alteration performed on paired tumor specimens is shown in the top row of each category. Subsequent rows illustrate the number of patients with conversed or changed (acquired or lost) status for given genetic alterations, specifying the number of patients who received systemic therapy (“S”) or irradiation (“I”) between surgeries

* One of these patients had conserved *IDH1* R132H mutations (Patient #33) and the other had conserved *IDH1* R132G mutations (Patient #32)

^#^Although this patient had conserved *ATRX* loss demonstrated on IHC, sequencing revealed different *ATRX* mutations in the diagnostic and recurrent tumor samples, as described in the text

^a^One of these patients had *CDKN2A* testing performed on three tumor specimens, with *CDKN2A* deletions identified (conserved) on the first two tumor samples, and subsequently lost on the third (post-mortem) tumor sample

**Table 3:** Comparison of patients whose tumors exhibited temporal genomic heterogeneity (n = 12) and patients whose tumors had completely conserved genomic profiles (n = 33)

	Patients whose tumors had temporal genomic heterogeneity	Patients whose tumors did not have temporal genomic heterogeneity	*p* value
n	12	33	–
Age at diagnosis [median (Range) in years]	5.8 (0.4–15.7)	6.1 (0.8–18.3)	0.70
Received systemic therapy and/or irradiation between surgeries [n (%)]	6 (50%)	14 (42%)	0.30
WHO grade I [n (%)]^a^	10 (91%)	24 (73%)	0.66

Statistics were performed using the Wilcoxon Rank-Sum test for differences in age, and using Fischer’s Exact test for differences in the proportions who received interval systemic therapy and/or irradiation and for WHO grade (I *versus* II) histology, with p-value < 0.05 considered significant

^a^Excluding patients with tumors of indeterminate WHO grading [pilomyxoid astrocytoma or low grade neoplasm (unclear if WHO grade I *versus* II)]

### Pilocytic/pilomyxoid astrocytomas

*BRAF* fusion or duplication: Of 23 patients with pilocytic or pilomyxoid astrocytomas who had testing for *BRAF* fusions or duplications performed on all paired surgical specimens, 15 (65%) tested positive at diagnosis and remained positive at recurrence (including nine patients who received systemic therapy [most with cytotoxic chemotherapy, three with MEK inhibitors (trametinib or selumetinib)] and one who received irradiation prior to subsequent surgery; Table 2). Testing for *BRAF* fusion or duplication remained negative in 8 (35%) patients. No acquisition or loss of *BRAF* fusions or duplications was identified in any patient.

*BRAF*^V600E^ mutation: Of 28 patients with pilocytic or pilomyxoid astrocytomas who had testing for *BRAF*^*V600E*^ mutations performed on all paired surgical specimens, 27 (96%) tested negative at diagnosis and remained negative at recurrence (including 14 patients who received systemic therapy and two who received irradiation prior to subsequent surgery; Table 2). One patient’s tumor tested positive by IHC at diagnosis, but lost this mutation at subsequent surgery 35 months after diagnosis (Patient #26, Fig. 1); this patient had not received previous systemic therapy or irradiation, and *BRAF*^V600E^ sequencing was not available for either specimen. Of note, 35 tumor samples from the entire cohort (spanning all histopathologic diagnoses) had *BRAF*^V600E^ testing performed by both IHC and targeted sequencing methods, and results were concordant in all but one [this patient with pilocytic astrocytoma had positive *BRAF*^V600E^ testing by IHC, but negative by sequencing, so this was interpreted as negative for the reported analysis (this patient’s subsequent tumor sample had negative *BRAF*^V600E^ IHC)].

*CDKN2A* deletion: Of 19 patients with pilocytic or pilomyxoid astrocytomas who had *CDKN2A* deletion testing performed on all paired surgical specimens, 13 (68%) were found to have a hemizygous deletion in at least one tumor sample, including several with low-level deletions (all above the testing laboratory- established threshold for positivity by FISH, requiring *CDKN2A* deletions in > 12% of 100 cells or > 11% of 200 cells). No homozygous deletions of *CDKN2A* were identified in this histologic subgroup or in the remainder of the cohort. *CDKN2A* deletion status was conserved in 10 (52%) patients with pilocytic or pilomyxoid astrocytomas, with four remaining positive and six remaining negative.

Three (16%) patients initially tested positive for *CDKN2A* deletion, then lost this genetic alteration on subsequent surgical resection. Two patients had not received systemic therapy or irradiation prior to subsequent surgery [Patients #18 and #26, Fig. 1 (Patient #26’s subsequent tumor specimen also lost prior *BRAF*^V600E^ mutation, as described above)]. The third patient had three tumor samples available for *CDKN2A* testing; *CDKN2A* deletions were conserved in the first two tumor samples (which were obtained 14 months apart and following chemotherapy), but no *CDKN2A* deletion was identified in the final specimen, obtained at autopsy approximately 7 years later and following further systemic therapy plus irradiation (Patient #5, Fig. 1).

Six (21%) patients acquired a *CDKN2A* deletion at recurrence or progression, including two patients who received cytotoxic chemotherapy (both with carboplatin and temozolomide, one with additional vincristine, vinblastine, and trametinib) and one who received photon irradiation prior to subsequent surgery (Table 4). Small (< 5%) increases in the Ki67 proliferative index were detected in three of the recurrent tumors which acquired *CDKN2A* deletions, compared to their respective diagnostic specimens lacking this alteration; no other concurrent unfavorable morphologic changes were identified with acquisition of *CDKN2A* deletion (i.e., no change in mitotic activity or WHO grade, as noted above).

**Table 4:** Clinical characteristics of patients whose tumors acquired *CDKN2A* deletions at recurrence/progression

Patient ID #	Age at diagnosis (years)	Sex	Tumor Pathologic classification	Tumor location	Metastatic disease at diagnosis	Extent of surgery #1	Time to first progression (months)	Time to surgery #2 (months)	Treatment received between diagnosis and gain of *CDKN2A* deletion	Current status (months from diagnosis to last follow-up)
1	5	F	Pilocytic astrocytoma	Diencephalic (hypothalamic/optic pathway)	No	STR	20	26	Chemotherapy (carboplatin, temozolomide)	Alive (104)
2	12	M	Pilocytic astrocytoma	Diencephalic (hypothalamic/optic pathway)	No	STR	2	27	Irradiation photon (50.4 Gy)]	Alive (116)
4	0.8	F	Pilomyxoid astrocytoma	Diencephalic (hypothalamic)	No	Biopsy	3.5	7	Chemotherapy (carboplatin/vincristine, vinblastine, temozolomide, trametinib), additional debulking surgery	Alive (58)
15	1	M	Pilocytic astrocytoma	Posterior fossa	No	STR	19	19	Surgery only	Alive (93)
18	5	M	Pilocytic astrocytoma	Posterior fossa	No	STR	17	17	Surgery only	Alive (182)
26	15	M	Pilocytic astrocytoma	Cerebrum (Right temporal-parietal)	No	STR (biopsy, then STR 1 week later)	5.5	5.5	Surgery only	Deceased (91; Drug overdose, unrelated to disease)
41	0.4	F	Desmoplastic infantile ganglioglioma	Cerebrum (Right frontal)	No	STR	4	8	Chemotherapy (carboplatin/vincristine)	Alive (94)

*H3K27M* mutation: Testing for the *H3K27M* mutation was performed on paired tumor samples from 13 patients with pilocytic or pilomyxoid astrocytomas (mostly with midline tumor locations) and was negative in all, including six patients who received systemic therapy and one who received irradiation prior to subsequent surgery (Table 2).

*FGFR1* rearrangement: An *FGFR1* rearrangement was detected in one of 19 (5%) patients with pilocytic or pilomyxoid astrocytomas who had this testing performed on all paired surgical specimens. This patient acquired an *FGFR1*-*TACC1* rearrangement at recurrence (initially tested negative), with no prior systemic therapy or irradiation (Patient #22, Fig. 1). The remaining 18 patients had conserved negative *FGFR1* rearrangement status on both primary and subsequent surgical specimens (including eight who received systemic therapy and two who received irradiation; Table 2).

*IDH1* mutations, *NTRK2*, *MYB*, and *MYBL1* rearrangements, and *ATRX* loss: Among patients with pilocytic or pilomyxoid astrocytomas who had testing for *IDH1* mutations (n = 15), rearrangements of *NTRK2* (n = 9), *MYB* (n = 10), or *MYBL1* (n = 7), and *ATRX* loss (n = 10) performed on both paired surgical specimens, results remained negative in all paired samples, with no acquisitions or losses, including after systemic therapy or irradiation (Table 2).

Additional targeted sequencing results: Targeted sequencing was performed on paired tumor specimens of 10 patients with pilocytic or pilomyxoid astrocytomas. No loss or acquisition of alterations in the following genes were identified over time or following treatment (including five patients who received systemic therapy prior to subsequent surgery): *AKT1, ALK, ATM, CDH1, CSF1R, CTNNB1, EGFR, ERBB2, ERBB4, EZH2, FBXW7, FGFR2, FGFR3, FLT3, GNA11, GNAQ, GNAS, HNF1A, HRAS, IDH2, JAK2, JAK3, KDR, KIT, KRAS, MLH1, MPL, NOTCH1, NPM1, NRAS, PDGFRA, PIK3CA, PTEN, PTPN11, RB1, RET, SMAD4, SMARCB1, SMO, SRC,* and *STK11*. A mutation in *EGFR* (N115K c.345T > A) was identified in one patient (Patient #19) and conserved at second surgery.

### Diffuse astrocytomas

*BRAF* fusion or duplication and *BRAF*^V600E^ mutation: Among patients with diffuse astrocytomas who had testing for *BRAF* fusion/duplication (n = 3) or *BRAF*^V600E^ mutation (n = 6) performed on all paired surgical specimens, testing remained negative in all, with no acquisition or loss (including in one patient who received systemic therapy and one patient who received irradiation prior to subsequent surgery; Table 2).

*IDH1* mutation and *ATRX* loss: Among seven patients with diffuse astrocytomas who had testing for *IDH1* mutations performed on paired surgical specimens, two (29%) patients tested positive at diagnosis and remained positive at recurrence. One patient (who received irradiation) had conserved *IDH1* R132H mutations identified by IHC (Patient #33, Fig. 1) and one patient had conserved *IDH1* R132G mutations identified on targeted sequencing (Patient #32, Fig. 1). *IDH1* mutation testing for the other five patients remained negative, with no acquisition or loss (Table 2).

Other temporal genomic changes (*CDKN2A* deletion, *ATRX* loss, *TP53* mutations) in one patient with an *IDH1*-mutant diffuse astrocytoma: The aforementioned patient with an *IDH1*-mutant (R132H) diffuse astrocytoma (Patient #33, Fig. 1) was found to have several genetic changes at recurrence and following radiotherapy, including loss of a hemizygous *CDKN2A* deletion (initially tested positive). Although conserved *ATRX* loss was demonstrated by IHC in both tumor samples, targeted sequencing revealed different *ATRX* mutations (R1426* in the diagnostic specimen and R1302fs*44 in the recurrent, post-irradiation specimen). Additionally, two somatic mutations in *TP53* (E258G, R267W) were detected at diagnosis and conserved at recurrence; however, additional unique *TP53* alterations were identified in this patient’s tumor samples, which were not shared (del exons 2–4, K132Q, N131del, R248W in the primary diagnostic sample, and R273C and E285* [both subclonal] in the recurrent sample), indicating possible loss and acquisition of these aberrations, respectively.

*H3K27M* mutation: *H3K27M* mutation testing was performed on paired tumor samples from five patients with diffuse astrocytomas and was negative in all, including one patient who received systemic therapy prior to subsequent surgery (Table 2).

*FGFR1, NTRK2*, *MYB*, and *MYBL1* rearrangements: Among patients with diffuse astrocytomas who had testing for rearrangements of *FGFR1* (n = 5), *NTRK2* (n = 4), *MYB* (n = 2), or *MYBL1* (n = 1) performed on both paired surgical specimens, results remained negative in all pairs, with no acquisition or loss (Table 2).

### Gangliogliomas

*BRAF* fusion or duplication: Testing for *BRAF* fusions or duplications was performed on paired surgical specimens in four patients with gangliogliomas, with conserved findings in all. Testing remained positive in two (50%) patients (including one who received systemic therapy prior to subsequent surgery) and remained negative in two (50%) patients (Table 2).

*BRAF*^V600E^ mutation: All five patients with gangliogliomas had *BRAF*^*V600E*^ mutation testing performed on paired surgical specimens, with no acquisition or loss detected. Two patients (40%) tested positive at diagnosis and remained positive at recurrence, and three patients (60%) remained negative (Table 2).

### Other LGGs

Paired tumor samples from five additional patients with LGGs with other histologic diagnoses were included for analysis [desmoplastic infantile ganglioglioma (n = 1), pleomorphic xanthoastrocytoma (n = 1), angiocentric glioma (n = 1), and low-grade glial or glioneuronal neoplasms, not otherwise specified (n = 2)]. Acquisition of a hemizygous *CDKN2A* deletion was identified in the patient with a desmoplastic infantile ganglioglioma following systemic therapy (Patient #41, Fig. 1 and Table 4). No other loss or gain of genetic alterations were detected in this patient or the other four patients by FISH, mutation-specific IHC, or targeted sequencing. Mutations in *CDH1* (A298T) and *FGFR1* (K656_T658 > MTP) were identified by targeted sequencing in one patient with a low-grade glioneuronal neoplasm (dysembryoplastic neuroepithelial tumor [DNET]-like) and were conserved at recurrence (Patient #45, Fig. 1).

### Prognostic impact of temporal changes in CDKN2A deletion status

**Fig. 3:**
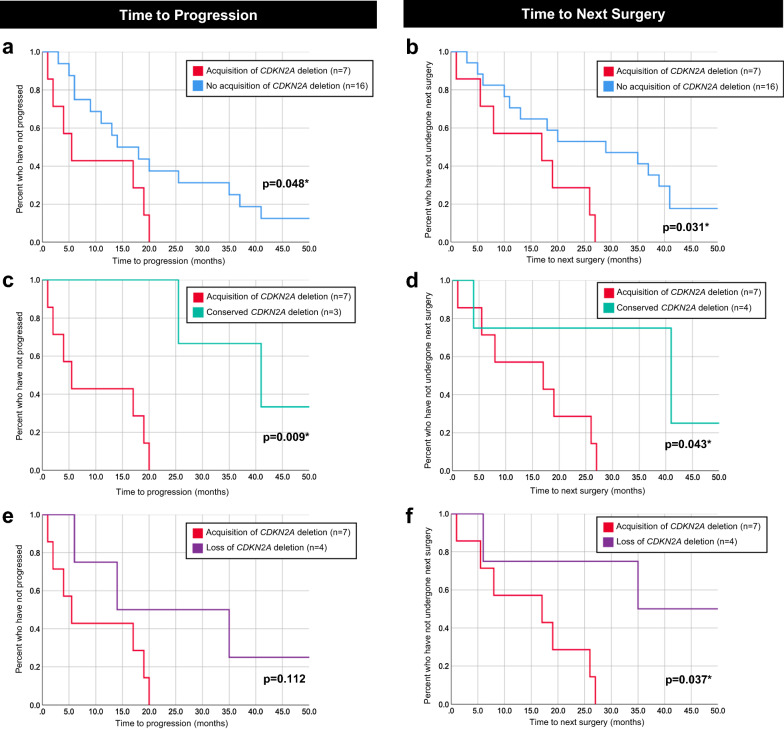
Kaplan Meier curves illustrating associations between temporal *CDKN2A* deletion status and time to progression (**a**, **c**, **e**) or time to next surgery (**b**, **d**, **f**) among patients who had *CDKN2A* deletion testing performed on paired tumor specimens. Shown are comparisons between patients whose tumors acquired *CDKN2A* deletions *versus* patients whose tumors did not acquire this genetic alteration (**a**, **b**), *versus* patients whose tumors had conserved *CDKN2A* deletions from diagnosis (**c**, **d**), and *versus* patients whose tumors lost *CDKN2A* deletions (**e**, **f**)

Among 24 patients in the entire cohort who had *CDKN2A* deletion testing performed on paired tumor specimens, shorter time to progression (defined as time from diagnosis to a new medical or surgical intervention in response to clinical and/or radiographic concern for progression) and shorter time to subsequent surgery were observed among the seven patients with acquired *CDKN2A* deletions compared to patients without acquisition of this genetic alteration (median time to progression: 5.5 months *versus* 16.0 months (*p* = 0.048); median time to next surgery: 17.0 months *versus* 29.0 months (*p* = 0.031), Fig. 3; note: patients without clinical or radiographic progression were excluded from this analysis). Additionally, patients whose tumors acquired *CDKN2A* deletions had shorter time to progression and shorter time to subsequent surgery compared to patients with conserved *CDKN2A* deletions on primary and recurrent tumor samples (median time to progression: 5.5 *versus* 41.0 months (*p* = 0.009); median time to next surgery: 17.0 *versus* 41.0 months (*p* = 0.043), Fig. 3). Acquisition of *CDKN2A* deletion was also associated with shorter time to subsequent surgery when compared to loss of *CDKN2A* deletion (median: 17.0 months *versus* 46.5 months (*p* = 0.037)].

### Genomic conservation in small sample of metastatic lesions

Four of 45 (9%) patients had metastatic disease at diagnosis and/or recurrence/progression. Two of these patients developed metastases at the time of recurrence, and surgical resection of these new metastatic lesions revealed identical genomic profiles to the respective primary tumors. One patient with a hypothalamic pilomyxoid astrocytoma developed tumor recurrence along a previous left frontal biopsy tract, which was subsequently biopsied (20 months after diagnostic surgery and following successive treatment with chemotherapy, a MEK inhibitor (trametinib), and an mTOR inhibitor); both primary and recurrent metastatic specimens remained positive for *BRAF* fusion and negative for *BRAF*^V600E^ (Patient #7, Fig. 1). One patient with a periventricular low-grade glioneuronal neoplasm (DNET-like) later developed a new, non-continuous right temporal lobe mass, which was subsequently resected (13 months after diagnostic surgery and following treatment with craniospinal irradiation); both primary and recurrent metastatic specimens were found to have the same aforementioned mutations in *CDH1* (A298T) and *FGFR1* (K656_T658 > MTP) on sequencing and were otherwise negative for *BRAF, FGFR1*, *NTRK2*, *MYB*, or *MYBL1* rearrangements, *CDKN2A* deletions, or *BRAF*^V600E^ or *IDH1* mutations (Patient #45, Fig. 1).

## Discussion

To our knowledge, this the first study to evaluate temporal and therapy-related genomic heterogeneity of pediatric LGGs through paired FISH, mutation-specific IHC, and/or targeted sequencing of a cohort of 94 total primary diagnostic and subsequent surgical tumor specimens. A direct comparison of the genomic profiles of paired samples reveals conservation of most genetic alterations over time and after therapy, but with possible changes in *CDKN2A* deletion status, including acquired *CDKN2A* deletions in a potentially higher risk subset of patients.

Most targetable genetic aberrations in pediatric LGGs, including *BRAF* alterations, are conserved over time, at recurrence, and following treatment with chemotherapy, other systemic therapy, or irradiation. These results are consistent with and significantly expand upon whole-genome sequencing data reported by Zhang et al. showing identical genetic profiles of two pairs of primary and recurrent pediatric LGGs, including preserved *FGFR1* duplications [68]. Importantly, *BRAF* fusion or duplication status was conserved in 100% of patients in our pediatric LGG cohort, with no acquisition or loss over time (including at third surgery) or after therapy. As *BRAF* fusions are identified in a large portion of pilocytic astrocytomas [29, 54] and there is growing evidence supporting the efficacy and tolerability of MEK inhibitors for pediatric LGGs harboring *BRAF* fusions [5, 8, 50], our findings have important therapeutic implications. Clinicians should feel confident that pediatric LGGs with *BRAF* fusions detected at diagnosis will retain this genetic alteration, such that targeted therapy with MEK inhibitors can be implemented at relapse without requiring genetic confirmation with repeat biopsy, in agreement with most providers’ current practice as well as previous clinical trials of these agents in the recurrent, refractory setting not mandating repeat molecular testing [5, 8, 50]. Additionally, *BRAF* fusions were conserved in recurrent tumor samples from three patients who progressed despite prior single-agent MEK inhibitor treatment, indicating likely preservation of this alteration following failed MEK inhibitor monotherapy. Although limited by a small sample size and deserving further exploration, this finding supports prior reports implicating alternative escape mechanisms (PI3K-AKT-mTOR signaling cascade), rather than loss of this *BRAF* alteration, in fusion-positive pediatric LGGs which develop resistance to MEK inhibitors, suggesting a potential role for future combination therapy in these patients [26].

*BRAF*^V600E^ mutations have been identified in certain subtypes of pediatric LGGs [17, 68] and also represent a promising therapeutic target, given emerging efficacy and safety data of BRAF inhibitors in children with recurrent LGGs harboring this alteration [6, 23, 34]. In our paired LGG cohort, *BRAF*^V600E^ mutation status (mostly negative) was conserved over time and following treatment in 98% of patients. Loss of *BRAF*^V600E^ was observed in one patient by IHC testing, though confirmatory sequencing was not available; while mutant-specific IHC for *BRAF*^V600E^ generally correlates well with *BRAF* sequencing, it may on occasion yield false positive or false negative results [18]. Furthermore, this patient did not receive previous systemic therapy or irradiation, and there was no obvious radiographic evidence of progression at the time of second surgery [performed due to clinical concern (increased seizures)], so this genetic change should be interpreted cautiously and may be the result of sampling bias. Our findings overall suggest conservation of *BRAF*^V600E^ mutation status in pediatric LGGs, including after previous therapy, yet with possible rare risk of loss that deserves further investigation.

Similarly, no tumors acquired *NTRK2*, *MYB*, or *MYBL1* fusions or *IDH1* mutations, suggesting preserved negative status of these genetic alterations can also likely be presumed at recurrence, including after prior treatment, without the need to obtain confirmatory biopsy tissue. Although acquisition of *FGFR1* rearrangement occurred in one patient (without preceding systemic therapy or irradiation), the majority (96%) of tumors with available *FGFR1* testing remained negative at recurrence and following therapy, supporting conserved status of this alteration commonly as well.

Histologic diagnosis and tumor grade were conserved in 100% of pediatric LGGs at recurrence or progression in our cohort, with no evidence of malignant transformation to HGG. Additionally, IHC testing for the *H3K27M* mutation, a genetic alteration which confers a dismal prognosis [32, 37] and is now sufficient criteria alone for WHO histologic grade IV diagnosis in patients with diffuse midline glioma [36], was negative in all tested paired tumor specimens, with no acquisition identified at time of relapse. These findings are consistent with prior studies suggesting evolution to HGG is extremely rare among pediatric LGGs even after prior systemic therapy or irradiation [9, 40].

A comparison of the molecular biology of primary and metastatic disease in pediatric LGGs is almost entirely absent in the literature to date [12]. Metastases at diagnosis and/or progression were seen in four (9%) patients in our cohort, consistent with a relatively low frequency of metastatic disease in pediatric LGGs [12]. Two of these metastatic lesions were biopsied or resected at the time of progression, and their genomic profiles were identical to the respective primary tumors, following chemotherapy in one patient and irradiation in the other patient. These results suggest genetic alterations may be conserved in metastases over time and after prior treatment; however, given the small number of patients with metastatic disease available for genomic testing, further study assessing spatial genomic heterogeneity in pediatric LGGs is needed to draw more definitive conclusions.

Although most genetic alterations were conserved over time in our pediatric LGG cohort, changes in *CDKN2A* deletion status at recurrence or progression were observed in 11 patients (42% of patients who had this testing performed on paired tumor specimens), with acquisition of hemizygous *CDKN2A* deletions in seven patients and loss in four. It is possible this discordance in *CDKN2A* deletion results over time is due to differences in sampling locations within tumor specimens and/or between tumor and closely surrounding or contaminated normal brain tissue. While we cannot definitively rule out sampling bias as an explanation for these findings, we believe this is less likely, given the lack of spatial heterogeneity of other genetic alterations analyzed, conservation of negative *CDKN2A* deletion status in one patient who had this testing performed on paired metastatic tumor samples, and evidence of spatial preservation of *CDKN2A* deletions from intratumoral genomic studies in glioblastoma [42, 56]. This genetic alteration has potential implications for treatment, as *CDKN2A* deletion or inactivation, which results in unrestricted progression through the G1-S cell cycle checkpoint, can be targeted with CDK4/6 inhibition. There is emerging evidence supporting the efficacy of CDK4/6 inhibitors in various solid tumors which harbor *CDKN2A* deletions [21, 55, 61], and there is growing clinical data suggesting adequate CNS penetration of ribociclib in studies evaluating its use in HGGs [15, 16, 59]. Given future potential for targeted therapy with a CDK4/6 inhibitor in the subset of pediatric LGGs which harbor *CDKN2A* deletions, repeating a biopsy at recurrence may be worthwhile in certain patients to evaluate for acquisition or loss of this actionable genetic alteration.

Among the seven patients in our cohort whose tumors gained hemizygous *CDKN2A* deletions, one had undergone photon irradiation, which has known DNA-damaging effects [52], three had received cytotoxic chemotherapy (all with carboplatin, two with vincristine and/or vinblastine, and two with temozolomide), and one was also treated with a MEK inhibitor. Temozolomide, an alkylating agent and thus a mutagen, has been associated with increased tumor mutational burden in adult LGGs at progression or malignant transformation [7, 11, 13, 27, 60, 62], but less is known about the impact of temozolomide therapy on the genomic evolution of biologically distinct pediatric gliomas. One study of paired pediatric HGGs found a trend toward increased number of mutations at recurrence in patients treated with temozolomide [51], but this did not reach statistical significance, and corresponding data in pediatric LGGs is lacking. While previous treatment with temozolomide or irradiation may contribute to risk of acquiring a *CDKN2A* deletion, three patients in our cohort gained this genetic alteration without any prior therapy and other patients treated with temozolomide or irradiation did not. Additionally, loss of actionable mutations at recurrence has been demonstrated in pediatric HGGs following targeted therapy [51], potentially as an acquired treatment resistance mechanism. Among the four patients in our cohort whose tumors exhibited loss of *CDKN2A* deletions, two had undergone previous irradiation. One of these patients was also treated with a MEK inhibitor, mTOR inhibitor, and lenalidomide in the time between previous surgery and autopsy; given the close interplay between the Ras/Raf/MEK/ERK, PI3K/Akt/mTOR, and CDK4/6/Rb pathways [45], with the former two acting upstream of the latter, it is possible that loss of *CDKN2A* deletion in this patient represented acquired resistance to prior MEK and mTOR inhibitor therapy. However, the other two patients with loss of *CDKN2A* deletions at recurrence had not been treated with prior systemic therapy or irradiation. Further research is therefore necessary to determine which tumors are at highest risk of acquiring or losing *CDKN2A* deletions, including investigating the role of prior treatment and whether temporal changes in *CDKN2A* deletion status contribute to acquired resistance to targeted agents.

Notably, shorter time to progression and subsequent surgery was observed in patients in our cohort whose tumors acquired hemizygous *CDKN2A* deletions, suggesting tumors that gain this genetic alteration may represent a unique subset of pediatric LGGs with a poorer prognosis. Previous studies have shown that the presence of *CDKN2A* deletions at diagnosis defines a higher risk group with worse outcomes [10, 35, 47, 53, 65]. Our novel findings indicate this genetic alteration can be acquired at relapse and may confer a worse prognosis. While conserved *CDKN2A* deletion status was not associated with poorer outcomes in our cohort (although interpretation is limited by the small sample size), patients with acquisition of hemizygous *CDKN2A* deletion at recurrence exhibited shorter time to progression and shorter time to next surgery, both when compared to patients without acquisition of this genetic alteration and when directly compared with patients whose tumors had preserved *CDKN2A* deletions from diagnosis [of note, there were no differences in other factors known to impact prognosis, including extent of first surgical resection (majority underwent subtotal resection) or proportion with diencephalic tumor location, between patients with gain of *CDKN2A* deletion *versus* those with conserved positive *CDKN2A* deletion status]. Acquired *CDKN2A* inactivation (gene deletion or hypermethylation resulting in decreased expression) at time of progression has been reported in studies of paired tumor analyses of other malignancies, including lymphoma, cervical cancer, and prostate cancer [19, 22, 46, 63]. Additionally, in two studies of pediatric LGGs which underwent malignant transformation, while *CDKN2A* loss was identified in a majority of tumors at diagnosis and conserved over time, two of 16 paired tumor specimens gained *CDKN2A* deletions at the time of evolution to HGG (one of eight from each report) [9, 40]. To our knowledge, this is the first study to suggest acquisition of *CDKN2A* deletion can occur and potentially contribute to progression in pediatric LGGs, even in the absence of malignant transformation.

It is important to acknowledge that all *CDKN2A* deletions identified in our cohort (with acquisition, loss, or conservation over time) were hemizygous, not homozygous. Although the presence of homozygous *CDKN2A* deletions at diagnosis in pediatric LGGs is well-recognized as an independent driver of cellular proliferation, with associated worse outcomes as described above [47, 53, 65], research into the biological consequences and prognostic impact of hemizygous deletions is lacking. Given reports demonstrating poorer prognosis in pediatric LGGs with decreased expression of p16^INF4a^ (protein encoded by *CDKN2A*) [25, 47], significant reduction in p16^INF4a^ expression among tumors with either hemizygous or homozygous *CDKN2A* deletions in at least one study [43], and aggressive clinical behavior of LGGs harboring hemizygous *CDKN2A* deletions [66], it is possible that the hemizygous *CDKN2A* deletions acquired in our cohort resulted in sufficiently low protein expression to drive cell cycle progression. However, another study conversely did not observe a clinically meaningful decrease in p16^INF4a^ expression in LGGs with hemizygous *CDKN2A* deletions [20]; therefore, the extent to which hemizygous *CDKN2A* deletions contribute to tumorigenesis, either independently or in combination with other genetic alterations, remains uncertain and critically deserving of further investigation. Given reported simultaneous acquisition of *CDKN2A* inactivation with other oncogenic mutations in solid tumors at progression [22] as well as genetic/epigenetic alterations in oncogenes or tumor suppressor genes commonly co-occurring with *CDKN2A* inactivation across various malignancies [57, 69], it is possible that pediatric LGGs which acquire hemizygous *CDKN2A* deletions have developed additional co-driver mutations, which were not tested for here, but should be investigated in future study. Additionally, there is emerging pre-clinical and clinical data explaining potential mechanisms by which *CDKN2A* deletions, perhaps in combination with other concurrent genetic aberrations, contribute to tumor progression in pediatric LGGs. Specifically, the role of *CDKN2A* inactivation in allowing escape from tumor senescence has been documented in pediatric LGGs [25, 35], and results from a pediatric LGG xenograft murine model suggests that *CDKN2A* deletion, in combination with *BRAF*^V600E^ mutation, is a key molecular change that mediates tumor progression, invasion, and migration [33]. Of the seven patients in our cohort whose tumors acquired hemizygous *CDKN2A* deletions, none had concurrent *BRAF*^V600E^ mutations at diagnosis or progression, but four had *BRAF* fusions which were conserved over time; further research is thus needed to determine whether hemizygous *CDKN2A* loss and *BRAF* fusions may act synergistically in facilitating tumor growth.

Lastly, in addition to loss of *CDKN2A* deletion, temporal changes in the *TP53* and *ATRX* genetic landscape of one adolescent patient with an *IDH1*-mutant diffuse astrocytoma, treated initially with radiotherapy alone, were identified at recurrence. Although some *TP53* mutations were conserved, both loss and acquisition of other likely pathogenic *TP53* genetic alterations occurred. Additionally, sequencing of the diagnostic and recurrent tumor specimens revealed different *ATRX* mutations, though both resulted in conserved loss of ATRX function. Irradiation-induced mutagenesis of *TP53* and *ATRX* has been reported, at least in secondary gliomas which developed following therapeutic irradiation for a prior malignancy [38]. Furthermore, in the aforementioned studies of paired pediatric HGGs and malignantly-transformed adult LGGs, heterogeneous genetic alterations of *TP53* and/or *ATRX* were similarly observed within the *IDH1*-mutant tumor pairs [27, 51], with conservation of *IDH1*-R132H mutations, similar to our patient. These findings deserve further exploration and may be more applicable to the adolescent and young adult neuro-oncology population, given the relatively low frequency of *IDH1*-mutations in pediatric LGGs [3, 64], which was re-demonstrated in our cohort.

Our study was limited by a small sample size and insufficient tumor tissue to perform all relevant molecular tests on all paired specimens, further decreasing the number of analyzable cases. Additional valuable genomic analyses such as whole genome and/or whole exome sequencing, RNA sequencing, and methylation testing were not available, and should therefore be incorporated into future studies in order to expand the molecular knowledge gained from FISH, mutation-specific IHC, and targeted sequencing performed here. Further research assessing spatial genomic heterogeneity in pediatric LGGs will also be critical, both to explore on a larger scale the above findings suggesting preservation of genetic alterations in metastases and to investigate sampling bias as a potential explanation for the discordance observed in *CDKN2A* deletion status, which we cannot definitely rule out. Finally, future investigation into potential mechanisms underlying acquisition or loss of *CDKN2A* deletions, risk factors for these temporal changes, and the biological consequences and prognostic impact of hemizygous (as opposed to homozygous) *CDKN2A* deletions, is essential.

Despite these limitations, this report characterizes temporal genomic heterogeneity in a pediatric LGG cohort and offers novel findings with important therapeutic implications. We demonstrate that most actionable genetic alterations in pediatric LGGs, including *BRAF* fusions or mutations, are conserved at recurrence, after prior systemic therapy or irradiation treatment, and in a small number of tumors with metastases. Repeat biopsy therefore is likely not necessary to confirm preservation of *BRAF* alteration status. Histologic diagnosis and grade remained the same in all tumors, with no acquisition of *H3K27M* mutations or evidence of malignant transformation. However, changes in *CDKN2A* deletion status over time were demonstrated, and acquisition of hemizygous *CDKN2A* deletion may define a higher risk subgroup of pediatric LGGs with a poorer prognosis. Given the potential for targeted therapies for tumors harboring *CDKN2A* deletions, performing a biopsy at recurrence may be indicated in certain patients, especially those with rapid progression.

## References

[1] (1988) A study of childhood brain tumors based on surgical biopsies from ten North American institutions: sample description. Childhood Brain Tumor Consortium. J Neurooncol 6:9–23. 10.1007/BF00163535 10.1007/BF00163535 3294353

[2] Ater JL, Zhou T, Holmes E, Mazewski CM, Booth TN, Freyer DR, Lazarus KH, Packer RJ, Prados M, Sposto R, Vezina G, Wisoff JH, Pollack IF (2012). Randomized study of two chemotherapy regimens for treatment of low-grade glioma in young children: a report from the Children’s Oncology Group. J Clin Oncol.

[3] Balss J, Meyer J, Mueller W, Korshunov A, Hartmann C, von Deimling A (2008). Analysis of the IDH1 codon 132 mutation in brain tumors. Acta Neuropathol.

[4] Bandopadhayay P, Ramkissoon LA, Jain P, Bergthold G, Wala J, Zeid R, Schumacher SE, Urbanski L, O’Rourke R, Gibson WJ, Pelton K, Ramkissoon SH, Han HJ, Zhu Y, Choudhari N, Silva A, Boucher K, Henn RE, Kang YJ, Knoff D, Paolella BR, Gladden-Young A, Varlet P, Pages M, Horowitz PM, Federation A, Malkin H, Tracy AA, Seepo S, Ducar M, Van Hummelen P, Santi M, Buccoliero AM, Scagnet M, Bowers DC, Giannini C, Puget S, Hawkins C, Tabori U, Klekner A, Bognar L, Burger PC, Eberhart C, Rodriguez FJ, Hill DA, Mueller S, Haas-Kogan DA, Phillips JJ, Santagata S, Stiles CD, Bradner JE, Jabado N, Goren A, Grill J, Ligon AH, Goumnerova L, Waanders AJ, Storm PB, Kieran MW, Ligon KL, Beroukhim R, Resnick AC (2016). MYB-QKI rearrangements in angiocentric glioma drive tumorigenicity through a tripartite mechanism. Nat Genet.

[5] Banerjee A, Jakacki RI, Onar-Thomas A, Wu S, Nicolaides T, Young Poussaint T, Fangusaro J, Phillips J, Perry A, Turner D, Prados M, Packer RJ, Qaddoumi I, Gururangan S, Pollack IF, Goldman S, Doyle LA, Stewart CF, Boyett JM, Kun LE, Fouladi M (2017). A phase I trial of the MEK inhibitor selumetinib (AZD6244) in pediatric patients with recurrent or refractory low-grade glioma: a Pediatric Brain Tumor Consortium (PBTC) study. Neuro Oncol.

[6] Bavle A, Jones J, Lin FY, Malphrus A, Adesina A, Su J (2017). Dramatic clinical and radiographic response to BRAF inhibition in a patient with progressive disseminated optic pathway glioma refractory to MEK inhibition. Pediatr Hematol Oncol.

[7] Bodell WJ, Gaikwad NW, Miller D, Berger MS (2003). Formation of DNA adducts and induction of lacI mutations in Big Blue Rat-2 cells treated with temozolomide: implications for the treatment of low-grade adult and pediatric brain tumors. Cancer Epidemiol Biomark Prev.

[8] Bouffet E, Kieran M, Hargrave D, Roberts S, Aerts I, Broniscer A, Geoerger B (2018). Trametinib therapy in pediatric patients with low-grade gliomas (LGG) with BRAF gene fusion; disease-specific cohort in the first pediatric testing of trametinib. Neuro Oncol.

[9] Broniscer A, Baker SJ, West AN, Fraser MM, Proko E, Kocak M, Dalton J, Zambetti GP, Ellison DW, Kun LE, Gajjar A, Gilbertson RJ, Fuller CE (2007). Clinical and molecular characteristics of malignant transformation of low-grade glioma in children. J Clin Oncol.

[10] Cagney DN, Miller MB, Dubuc A, Delalle I, Ligon AH, Chukwueke U, Al-Mefty O, Aizer A, Ligon K, Wen P (2019). Clinical importance of CDKN2A loss and monosomy 10 in pilocytic astrocytoma. Cureus.

[11] Campbell BB, Light N, Fabrizio D, Zatzman M, Fuligni F, de Borja R, Davidson S, Edwards M, Elvin JA, Hodel KP, Zahurancik WJ, Suo Z, Lipman T, Wimmer K, Kratz CP, Bowers DC, Laetsch TW, Dunn GP, Johanns TM, Grimmer MR, Smirnov IV, Larouche V, Samuel D, Bronsema A, Osborn M, Stearns D, Raman P, Cole KA, Storm PB, Yalon M, Opocher E, Mason G, Thomas GA, Sabel M, George B, Ziegler DS, Lindhorst S, Issai VM, Constantini S, Toledano H, Elhasid R, Farah R, Dvir R, Dirks P, Huang A, Galati MA, Chung J, Ramaswamy V, Irwin MS, Aronson M, Durno C, Taylor MD, Rechavi G, Maris JM, Bouffet E, Hawkins C, Costello JF, Meyn MS, Pursell ZF, Malkin D, Tabori U, Shlien A (2017). Comprehensive analysis of hypermutation in human cancer. Cell.

[12] Chamdine O, Broniscer A, Wu S, Gajjar A, Qaddoumi I (2016). Metastatic low-grade gliomas in children: 20 years’ experience at St. Jude Children’s Research Hospital. Pediatr Blood Cancer.

[13] Choi S, Yu Y, Grimmer MR, Wahl M, Chang SM, Costello JF (2018). Temozolomide-associated hypermutation in gliomas. Neuro Oncol.

[14] de Blank P, Bandopadhayay P, Haas-Kogan D, Fouladi M, Fangusaro J (2019). Management of pediatric low-grade glioma. Curr Opin Pediatr.

[15] DeWire M, Fuller C, Hummel T, Chow L, Salloum R, Pater L (2017). CLEE011XUS17T (NCT 02607124): a Phase I/II study of ribociclib (lee011) following radiation therapy in children with newly diagnosed non-biopsied diffuse pontine gliomas (dipg) and Rb + biopsied dipg and high grade gliomas (HGG. Neuro-Oncology.

[16] Dewire M, Fuller C, Hummel T, Chow L, Salloum R, Pater L (2018). DIPG-73. CLEE011XUS17T (NCT 02607124): a phase I/II study of ribociclib (LEE011) following radiation therapy (RT) in children and young adults with newly diagnosed non-biopsied diffuse pontine gliomas (DIPG) and Rb + biopsied dipg and high grade gliomas (HGG). Neuro-Oncology.

[17] Dias-Santagata D, Lam Q, Vernovsky K, Vena N, Lennerz JK, Borger DR, Batchelor TT, Ligon KL, Iafrate AJ, Ligon AH, Louis DN, Santagata S (2011). BRAF V600E mutations are common in pleomorphic xanthoastrocytoma: diagnostic and therapeutic implications. PLoS ONE.

[18] Dvorak K, Aggeler B, Palting J, McKelvie P, Ruszkiewicz A, Waring P (2014). Immunohistochemistry with the anti-BRAF V600E (VE1) antibody: impact of pre-analytical conditions and concordance with DNA sequencing in colorectal and papillary thyroid carcinoma. Pathology.

[19] Elenitoba-Johnson KS, Gascoyne RD, Lim MS, Chhanabai M, Jaffe ES, Raffeld M (1998). Homozygous deletions at chromosome 9p21 involving p16 and p15 are associated with histologic progression in follicle center lymphoma. Blood.

[20] Frazão L, do Carmo Martins M, Nunes VM, Pimentel J, Faria C, Miguéns J, Sagarribay A, Matos M, Salgado D, Nunes S, Mafra M, Roque L (2018). BRAF V600E mutation and 9p21: cDKN2A/B and MTAP co-deletions—markers in the clinical stratification of pediatric gliomas. BMC Cancer.

[21] Gopalan PK, Pinder MC, Chiappori A, Ivey AM, Villegas AG (2014). A phase II clinical trial of the CDK 4/6 inhibitor palbociclib (PD 0332991) in previously treated, advanced non-small cell lung cancer (NSCLC) patients with inactivated *CDKN2A*. J Clin Oncol.

[22] Han GC, Hwang J, Mullane SA, Cibulskis C, Zhang Z (2017). Abstract 2905: clinical and genomic resistance to second generation androgen blockade in paired biopsies of metastatic castration-resistant prostate cancer. Cancer Res.

[23] Hargrave D, Bouffet E, Tabori U, Broniscer A, Cohen K, Hansford J (2019). Efficacy and safety of dabrafenib in pediatric patients with BRAF V600 mutation-positive relapsed or refractory low-grade glioma: results from a phase I/IIa study. Clin Cancer Res.

[24] Hoffman LM, Donson AM, Nakachi I, Griesinger AM, Birks DK, Amani V, Hemenway MS, Liu AK, Wang M, Hankinson TC, Handler MH, Foreman NK (2014). Molecular sub-group-specific immunophenotypic changes are associated with outcome in recurrent posterior fossa ependymoma. Acta Neuropathol.

[25] Jacob K, Quang-Khuong DA, Jones DT, Witt H, Lambert S, Albrecht S, Witt O, Vezina C, Shirinian M, Faury D, Garami M, Hauser P, Klekner A, Bognar L, Farmer JP, Montes JL, Atkinson J, Hawkins C, Korshunov A, Collins VP, Pfister SM, Tabori U, Jabado N (2011). Genetic aberrations leading to MAPK pathway activation mediate oncogene-induced senescence in sporadic pilocytic astrocytomas. Clin Cancer Res.

[26] Jain P, Silva A, Han HJ, Lang SS, Zhu Y, Boucher K, Smith TE, Vakil A, Diviney P, Choudhari N, Raman P, Busch CM, Delaney T, Yang X, Olow AK, Mueller S, Haas-Kogan D, Fox E, Storm PB, Resnick AC, Waanders AJ (2017). Overcoming resistance to single-agent therapy for oncogenic BRAF gene fusions via combinatorial targeting of MAPK and PI3K/mTOR signaling pathways. Oncotarget.

[27] Johnson BE, Mazor T, Hong C, Barnes M, Aihara K, McLean CY, Fouse SD, Yamamoto S, Ueda H, Tatsuno K, Asthana S, Jalbert LE, Nelson SJ, Bollen AW, Gustafson WC, Charron E, Weiss WA, Smirnov IV, Song JS, Olshen AB, Cha S, Zhao Y, Moore RA, Mungall AJ, Jones SJM, Hirst M, Marra MA, Saito N, Aburatani H, Mukasa A, Berger MS, Chang SM, Taylor BS, Costello JF (2014). Mutational analysis reveals the origin and therapy-driven evolution of recurrent glioma. Science.

[28] Jones C, Perryman L, Hargrave D (2012). Paediatric and adult malignant glioma: close relatives or distant cousins?. Nat Rev Clin Oncol.

[29] Jones DT, Kocialkowski S, Liu L, Pearson DM, Bäcklund LM, Ichimura K, Collins VP (2008). Tandem duplication producing a novel oncogenic BRAF fusion gene defines the majority of pilocytic astrocytomas. Cancer Res.

[30] Jones DT, Mulholland SA, Pearson DM, Malley DS, Openshaw SW, Lambert SR, Liu L, Bäcklund LM, Ichimura K, Collins VP (2011). Adult grade II diffuse astrocytomas are genetically distinct from and more aggressive than their paediatric counterparts. Acta Neuropathol.

[31] Jones DTW, Kieran MW, Bouffet E, Alexandrescu S, Bandopadhayay P, Bornhorst M, Ellison D, Fangusaro J, Fisher MJ, Foreman N, Fouladi M, Hargrave D, Hawkins C, Jabado N, Massimino M, Mueller S, Perilongo G, Schouten van Meeteren AYN, Tabori U, Warren K, Waanders AJ, Walker D, Weiss W, Witt O, Wright K, Zhu Y, Bowers DC, Pfister SM, Packer RJ (2018). Pediatric low-grade gliomas: next biologically driven steps. Neuro Oncol.

[32] Karremann M, Gielen GH, Hoffmann M, Wiese M, Colditz N, Warmuth-Metz M, Bison B, Claviez A, van Vuurden DG, von Bueren AO, Gessi M, Kühnle I, Hans VH, Benesch M, Sturm D, Kortmann RD, Waha A, Pietsch T, Kramm CM (2018). Diffuse high-grade gliomas with H3 K27M mutations carry a dismal prognosis independent of tumor location. Neuro Oncol.

[33] Kogiso M, Qi L, Lindsay H, Huang Y, Zhao X, Liu Z, Braun FK, Du Y, Zhang H, Bae G, Zhao S, Injac SG, Sobieski M, Brunell D, Mehta V, Tran D, Murray J, Baxter PA, Yuan XJ, Su JM, Adesina A, Perlaky L, Chintagumpala M, Parsons DW, Lau CC, Stephan CC, Lu X, Li XN (2017). Xenotransplantation of pediatric low grade gliomas confirms the enrichment. Oncotarget.

[34] Lassaletta A, Guerreiro Stucklin A, Ramaswamy V, Zapotocky M, McKeown T, Hawkins C, Bouffet E, Tabori U (2016). Profound clinical and radiological response to BRAF inhibition in a 2-month-old diencephalic child with hypothalamic/chiasmatic glioma. Pediatr Blood Cancer.

[35] Lassaletta A, Zapotocky M, Mistry M, Ramaswamy V, Honnorat M, Krishnatry R, Guerreiro Stucklin A, Zhukova N, Arnoldo A, Ryall S, Ling C, McKeown T, Loukides J, Cruz O, de Torres C, Ho CY, Packer RJ, Tatevossian R, Qaddoumi I, Harreld JH, Dalton JD, Mulcahy-Levy J, Foreman N, Karajannis MA, Wang S, Snuderl M, Nageswara Rao A, Giannini C, Kieran M, Ligon KL, Garre ML, Nozza P, Mascelli S, Raso A, Mueller S, Nicolaides T, Silva K, Perbet R, Vasiljevic A, Faure Conter C, Frappaz D, Leary S, Crane C, Chan A, Ng HK, Shi ZF, Mao Y, Finch E, Eisenstat D, Wilson B, Carret AS, Hauser P, Sumerauer D, Krskova L, Larouche V, Fleming A, Zelcer S, Jabado N, Rutka JT, Dirks P, Taylor MD, Chen S, Bartels U, Huang A, Ellison DW, Bouffet E, Hawkins C, Tabori U (2017). Therapeutic and prognostic implications of BRAF V600E in pediatric low-grade gliomas. J Clin Oncol.

[36] Louis DN, Perry A, Reifenberger G, von Deimling A, Figarella-Branger D, Cavenee WK, Ohgaki H, Wiestler OD, Kleihues P, Ellison DW (2016). The 2016 World Health Organization classification of tumors of the central nervous system: a summary. Acta Neuropathol.

[37] Lu VM, Alvi MA, McDonald KL, Daniels DJ (2018). Impact of the H3K27M mutation on survival in pediatric high-grade glioma: a systematic review and meta-analysis. J Neurosurg Pediatr.

[38] López GY, Van Ziffle J, Onodera C, Grenert JP, Yeh I, Bastian BC, Clarke J, Oberheim Bush NA, Taylor J, Chang S, Butowski N, Banerjee A, Mueller S, Kline C, Torkildson J, Samuel D, Siongco A, Raffel C, Gupta N, Kunwar S, Mummaneni P, Aghi M, Theodosopoulos P, Berger M, Phillips JJ, Pekmezci M, Tihan T, Bollen AW, Perry A, Solomon DA (2019). The genetic landscape of gliomas arising after therapeutic radiation. Acta Neuropathol.

[39] Merchant TE, Conklin HM, Wu S, Lustig RH, Xiong X (2009). Late effects of conformal radiation therapy for pediatric patients with low-grade glioma: prospective evaluation of cognitive, endocrine, and hearing deficits. J Clin Oncol.

[40] Mistry M, Zhukova N, Merico D, Rakopoulos P, Krishnatry R, Shago M, Stavropoulos J, Alon N, Pole JD, Ray PN, Navickiene V, Mangerel J, Remke M, Buczkowicz P, Ramaswamy V, Guerreiro Stucklin A, Li M, Young EJ, Zhang C, Castelo-Branco P, Bakry D, Laughlin S, Shlien A, Chan J, Ligon KL, Rutka JT, Dirks PB, Taylor MD, Greenberg M, Malkin D, Huang A, Bouffet E, Hawkins CE, Tabori U (2015). BRAF mutation and CDKN2A deletion define a clinically distinct subgroup of childhood secondary high-grade glioma. J Clin Oncol.

[41] Morrissy AS, Garzia L, Shih DJ, Zuyderduyn S, Huang X, Skowron P, Remke M, Cavalli FM, Ramaswamy V, Lindsay PE, Jelveh S, Donovan LK, Wang X, Luu B, Zayne K, Li Y, Mayoh C, Thiessen N, Mercier E, Mungall KL, Ma Y, Tse K, Zeng T, Shumansky K, Roth AJ, Shah S, Farooq H, Kijima N, Holgado BL, Lee JJ, Matan-Lithwick S, Liu J, Mack SC, Manno A, Michealraj KA, Nor C, Peacock J, Qin L, Reimand J, Rolider A, Thompson YY, Wu X, Pugh T, Ally A, Bilenky M, Butterfield YS, Carlsen R, Cheng Y, Chuah E, Corbett RD, Dhalla N, He A, Lee D, Li HI, Long W, Mayo M, Plettner P, Qian JQ, Schein JE, Tam A, Wong T, Birol I, Zhao Y, Faria CC, Pimentel J, Nunes S, Shalaby T, Grotzer M, Pollack IF, Hamilton RL, Li XN, Bendel AE, Fults DW, Walter AW, Kumabe T, Tominaga T, Collins VP, Cho YJ, Hoffman C, Lyden D, Wisoff JH, Garvin JH, Stearns DS, Massimi L, Schüller U, Sterba J, Zitterbart K, Puget S, Ayrault O, Dunn SE, Tirapelli DP, Carlotti CG, Wheeler H, Hallahan AR, Ingram W, MacDonald TJ, Olson JJ, Van Meir EG, Lee JY, Wang KC, Kim SK, Cho BK, Pietsch T, Fleischhack G, Tippelt S, Ra YS, Bailey S, Lindsey JC, Clifford SC, Eberhart CG, Cooper MK, Packer RJ, Massimino M, Garre ML, Bartels U, Tabori U, Hawkins CE, Dirks P, Bouffet E, Rutka JT, Wechsler-Reya RJ, Weiss WA, Collier LS, Dupuy AJ, Korshunov A, Jones DT, Kool M, Northcott PA, Pfister SM, Largaespada DA, Mungall AJ, Moore RA, Jabado N, Bader GD, Jones SJ, Malkin D, Marra MA, Taylor MD (2016). Divergent clonal selection dominates medulloblastoma at recurrence. Nature.

[42] Nakajima N, Nobusawa S, Nakata S, Nakada M, Yamazaki T, Matsumura N, Harada K, Matsuda H, Funata N, Nagai S, Nakamura H, Sasaki A, Akimoto J, Hirato J, Yokoo H (2018). BRAF V600E, TERT promoter mutations and CDKN2A/B homozygous deletions are frequent in epithelioid glioblastomas: a histological and molecular analysis focusing on intratumoral heterogeneity. Brain Pathol.

[43] Nakamura M, Konishi N, Hiasa Y, Tsunoda S, Nakase H, Tsuzuki T, Aoki H, Sakitani H, Inui T, Sakaki T (1998). Frequent alterations of cell-cycle regulators in astrocytic tumors as detected by molecular genetic and immunohistochemical analyses. Brain Tumor Pathol.

[44] Packer RJ, Pfister S, Bouffet E, Avery R, Bandopadhayay P, Bornhorst M, Bowers DC, Ellison D, Fangusaro J, Foreman N, Fouladi M, Gajjar A, Haas-Kogan D, Hawkins C, Ho CY, Hwang E, Jabado N, Kilburn LB, Lassaletta A, Ligon KL, Massimino M, Meeteren SV, Mueller S, Nicolaides T, Perilongo G, Tabori U, Vezina G, Warren K, Witt O, Zhu Y, Jones DT, Kieran M (2017). Pediatric low-grade gliomas: implications of the biologic era. Neuro Oncol.

[45] Paternot S, Roger PP (2009). Combined inhibition of MEK and mammalian target of rapamycin abolishes phosphorylation of cyclin-dependent kinase 4 in glioblastoma cell lines and prevents their proliferation. Cancer Res.

[46] Pinyol M, Cobo F, Bea S, Jares P, Nayach I, Fernandez PL, Montserrat E, Cardesa A, Campo E (1998). p16(INK4a) gene inactivation by deletions, mutations, and hypermethylation is associated with transformed and aggressive variants of non-Hodgkin’s lymphomas. Blood.

[47] Raabe EH, Lim KS, Kim JM, Meeker A, Mao XG, Nikkhah G, Maciaczyk J, Kahlert U, Jain D, Bar E, Cohen KJ, Eberhart CG (2011). BRAF activation induces transformation and then senescence in human neural stem cells: a pilocytic astrocytoma model. Clin Cancer Res.

[48] Ramaswamy V, Remke M, Bouffet E, Faria CC, Perreault S, Cho YJ, Shih DJ, Luu B, Dubuc AM, Northcott PA, Schüller U, Gururangan S, McLendon R, Bigner D, Fouladi M, Ligon KL, Pomeroy SL, Dunn S, Triscott J, Jabado N, Fontebasso A, Jones DT, Kool M, Karajannis MA, Gardner SL, Zagzag D, Nunes S, Pimentel J, Mora J, Lipp E, Walter AW, Ryzhova M, Zheludkova O, Kumirova E, Alshami J, Croul SE, Rutka JT, Hawkins C, Tabori U, Codispoti KE, Packer RJ, Pfister SM, Korshunov A, Taylor MD (2013). Recurrence patterns across medulloblastoma subgroups: an integrated clinical and molecular analysis. Lancet Oncol.

[49] Ramkissoon LA, Horowitz PM, Craig JM, Ramkissoon SH, Rich BE, Schumacher SE, McKenna A, Lawrence MS, Bergthold G, Brastianos PK, Tabak B, Ducar MD, Van Hummelen P, MacConaill LE, Pouissant-Young T, Cho YJ, Taha H, Mahmoud M, Bowers DC, Margraf L, Tabori U, Hawkins C, Packer RJ, Hill DA, Pomeroy SL, Eberhart CG, Dunn IF, Goumnerova L, Getz G, Chan JA, Santagata S, Hahn WC, Stiles CD, Ligon AH, Kieran MW, Beroukhim R, Ligon KL (2013). Genomic analysis of diffuse pediatric low-grade gliomas identifies recurrent oncogenic truncating rearrangements in the transcription factor MYBL1. Proc Natl Acad Sci U S A.

[50] Robison N, Pauly J, Malvar J, Filbin M, Loret de Mola R, Dorris K, Bendel A, Bowers D (2017). A phase I dose escalation trial of the MEK1/2 inhibitor MEK162 (Binimetinib) in children with low-grade gliomas and other RAS-RAF pathway-activated tumors: initial report. Neuro-Oncology.

[51] Salloum R, McConechy MK, Mikael LG, Fuller C, Drissi R, DeWire M, Nikbakht H, De Jay N, Yang X, Boue D, Chow LML, Finlay JL, Gayden T, Karamchandani J, Hummel TR, Olshefski R, Osorio DS, Stevenson C, Kleinman CL, Majewski J, Fouladi M, Jabado N (2017). Characterizing temporal genomic heterogeneity in pediatric high-grade gliomas. Acta Neuropathol Commun.

[52] Sankaranarayanan K, Taleei R, Rahmanian S, Nikjoo H (2013). Ionizing radiation and genetic risks. XVII. Formation mechanisms underlying naturally occurring DNA deletions in the human genome and their potential relevance for bridging the gap between induced DNA double-strand breaks and deletions in irradiated germ cells. Mutat Res.

[53] Schiffman JD, Hodgson JG, VandenBerg SR, Flaherty P, Polley MY, Yu M, Fisher PG, Rowitch DH, Ford JM, Berger MS, Ji H, Gutmann DH, James CD (2010). Oncogenic BRAF mutation with CDKN2A inactivation is characteristic of a subset of pediatric malignant astrocytomas. Cancer Res.

[54] Sievert AJ, Jackson EM, Gai X, Hakonarson H, Judkins AR, Resnick AC, Sutton LN, Storm PB, Shaikh TH, Biegel JA (2009). Duplication of 7q34 in pediatric low-grade astrocytomas detected by high-density single-nucleotide polymorphism-based genotype arrays results in a novel BRAF fusion gene. Brain Pathol.

[55] Sobhani N, Corona SP, Zanconati F, Generali D (2017). Cyclin dependent kinase 4 and 6 inhibitors as novel therapeutic agents for targeted treatment of malignant mesothelioma. Genes Cancer.

[56] Sottoriva A, Spiteri I, Piccirillo SG, Touloumis A, Collins VP, Marioni JC, Curtis C, Watts C, Tavaré S (2013). Intratumor heterogeneity in human glioblastoma reflects cancer evolutionary dynamics. Proc Natl Acad Sci U S A.

[57] Tam KW, Zhang W, Soh J, Stastny V, Chen M, Sun H, Thu K, Rios JJ, Yang C, Marconett CN, Selamat SA, Laird-Offringa IA, Taguchi A, Hanash S, Shames D, Ma X, Zhang MQ, Lam WL, Gazdar A (2013). CDKN2A/p16 inactivation mechanisms and their relationship to smoke exposure and molecular features in non-small-cell lung cancer. J Thorac Oncol.

[58] Tatevossian RG, Tang B, Dalton J, Forshew T, Lawson AR, Ma J, Neale G, Shurtleff SA, Bailey S, Gajjar A, Baker SJ, Sheer D, Ellison DW (2010). MYB upregulation and genetic aberrations in a subset of pediatric low-grade gliomas. Acta Neuropathol.

[59] Tien A-C, Bao X, Derogatis A, Kim S, Mehta S (2018). ACTR-45 phase 0/2 study of ribociclib in patients with recurrent glioblastoma. Neuro-Oncology.

[60] Tom MC, Park DYJ, Yang K, Leyrer CM, Wei W, Jia X, Varra V, Yu JS, Chao ST, Balagamwala EH, Suh JH, Vogelbaum MA, Barnett GH, Prayson RA, Stevens GHJ, Peereboom DM, Ahluwalia MS, Murphy ES (2019). Malignant transformation of molecularly classified adult low-grade glioma. Int J Radiat Oncol Biol Phys.

[61] Tripathy D, Bardia A, Sellers WR (2017). Ribociclib (LEE011): mechanism of action and clinical impact of this selective cyclin-dependent kinase 4/6 inhibitor in various solid tumors. Clin Cancer Res.

[62] van Thuijl HF, Mazor T, Johnson BE, Fouse SD, Aihara K, Hong C, Malmström A, Hallbeck M, Heimans JJ, Kloezeman JJ, Stenmark-Askmalm M, Lamfers ML, Saito N, Aburatani H, Mukasa A, Berger MS, Söderkvist P, Taylor BS, Molinaro AM, Wesseling P, Reijneveld JC, Chang SM, Ylstra B, Costello JF (2015). Evolution of DNA repair defects during malignant progression of low-grade gliomas after temozolomide treatment. Acta Neuropathol.

[63] Wijetunga NA, Belbin TJ, Burk RD, Whitney K, Abadi M, Greally JM, Einstein MH, Schlecht NF (2016). Novel epigenetic changes in CDKN2A are associated with progression of cervical intraepithelial neoplasia. Gynecol Oncol.

[64] Yan H, Parsons DW, Jin G, McLendon R, Rasheed BA, Yuan W, Kos I, Batinic-Haberle I, Jones S, Riggins GJ, Friedman H, Friedman A, Reardon D, Herndon J, Kinzler KW, Velculescu VE, Vogelstein B, Bigner DD (2009). IDH1 and IDH2 mutations in gliomas. N Engl J Med.

[65] Yang RR, Aibaidula A, Wang WW, Chan AK, Shi ZF, Zhang ZY, Chan DTM, Poon WS, Liu XZ, Li WC, Zhang RQ, Li YX, Chung NY, Chen H, Wu J, Zhou L, Li KK, Ng HK (2018). Pediatric low-grade gliomas can be molecularly stratified for risk. Acta Neuropathol.

[66] Yeo YH, Byrne NP, Counelis GJ, Perry A (2013). Adult with cerebellar anaplastic pilocytic astrocytoma associated with BRAF V600E mutation and p16 loss. Clin Neuropathol.

[67] Yuan L, Chi Y, Chen W, Chen X, Wei P, Sheng W, Zhou X, Shi D (2015). Immunohistochemistry and microsatellite instability analysis in molecular subtyping of colorectal carcinoma based on mismatch repair competency. Int J Clin Exp Med.

[68] Zhang J, Wu G, Miller CP, Tatevossian RG, Dalton JD, Tang B, Orisme W, Punchihewa C, Parker M, Qaddoumi I, Boop FA, Lu C, Kandoth C, Ding L, Lee R, Huether R, Chen X, Hedlund E, Nagahawatte P, Rusch M, Boggs K, Cheng J, Becksfort J, Ma J, Song G, Li Y, Wei L, Wang J, Shurtleff S, Easton J, Zhao D, Fulton RS, Fulton LL, Dooling DJ, Vadodaria B, Mulder HL, Tang C, Ochoa K, Mullighan CG, Gajjar A, Kriwacki R, Sheer D, Gilbertson RJ, Mardis ER, Wilson RK, Downing JR, Baker SJ, Ellison DW, Project SJCsRHWUPCG (2013). Whole-genome sequencing identifies genetic alterations in pediatric low-grade gliomas. Nat Genet.

[69] Zhao R, Choi BY, Lee MH, Bode AM, Dong Z (2016). Implications of genetic and epigenetic alterations of CDKN2A (p16(INK4a)) in cancer. EBioMedicine.

